# Unmasking spatial disparities and survival bias in the United States diabetes prevalence: A multilevel analysis of individual and ecological determinants using the 2024 behavioral risk factor surveillance system

**DOI:** 10.1016/j.pmedr.2026.103688

**Published:** 2026-10-28

**Authors:** Masoud Imani, Seyedshayan Shojaei, Kimia Heidari, Mohammad Javad Shabani, Hamid Mohsenabadi, Bagher Larijani, Mohammad Reza Mohajeri-Tehrani, Amir Kasaeian

**Affiliations:** a Endocrinology and Metabolism Research Center, Endocrinology and Metabolism Clinical Sciences Institute, Tehran University of Medical Sciences, Tehran, Iran; b Liver and Pancreatobiliary Diseases Research Center, Digestive Diseases Research Institute, Tehran University of Medical Sciences, Tehran, Iran; c Western University of Health Sciences, College of Osteopathic Medicine of the Pacific, Pomona, California, USA; d University of California, Irvine, School of Medicine, Irvine, California, USA; e Department of Clinical Psychology, School of Behavioral Sciences and Mental Health (Tehran Institute of Psychiatry), Iran University of Medical Sciences, Tehran, Iran; f Research Center for Chronic Inflammatory Diseases, Tehran University of Medical Sciences, Tehran, Iran; g Digestive Oncology Research Center, Digestive Diseases Research Institute, Tehran University of Medical Sciences, Tehran, Iran

**Keywords:** Diabetes, Multilevel modeling, Epidemiology, Cross-sectional data, Behavioral risk factor surveillance system

## Abstract

**Objective:**

Diabetes prevalence varies across the United States, but individual versus state-level contributions remain unclear. We examined these predictors and survival bias.

**Methods:**

Using 2024 data from 406,782 adults in the United States Behavioral Risk Factor Surveillance System, we fitted multilevel logistic models for individual and state-level covariates. Age-stratified analyses tested survival bias.

**Results:**

Individual obesity was the strongest predictor (OR = 2.33; 95% CI: 2.28, 2.38). Asian adults exhibited the highest risk (OR = 2.05; 95% CI: 1.91, 2.20), followed by Black (OR = 1.76) and Hispanic adults (OR = 1.54). State-level obesity independently predicted diabetes (OR = 1.38 per 10-point increase; 95% CI: 1.17, 1.63), explaining 57% of between-state variance. Individual obesity effects attenuated in higher-obesity states. Inverse associations for smoking (OR = 0.89; 95% CI: 0.86, 0.92) and binge drinking (OR = 0.62; 95% CI: 0.60, 0.65) were null in young adults but strengthened with age.

**Conclusions:**

State-level context independently elevates diabetes risk. Asian Americans face disproportionately high risk, supporting lower body mass index screening thresholds. Apparent protective effects of smoking and binge drinking reflect survival bias.

## 1 Introduction

Diabetes mellitus continues to be a central public health challenge in the United States, driving high rates of illness, early mortality, and severe healthcare strain (Harding et al., 2025). Because type 2 diabetes accounts for the vast majority of adult cases, public health efforts heavily focus on modifiable metabolic factors. While body mass index (BMI) remains the primary measure for individual adiposity and diabetes risk (Ong et al., 2023), relying strictly on individual markers falls short when explaining why diabetes rates vary so drastically across different regions and demographic groups.

A major complication in clinical risk assessment is that standard obesity metrics do not apply uniformly across racial and ethnic backgrounds. The heavy diabetes burden in Black and Hispanic communities is closely linked to socioeconomic marginalization and higher overall obesity rates (Hassan et al., 2023). Furthermore, epidemiological data for Asian Americans reveal a unique paradox. Studies demonstrate that individuals of Asian descent are highly susceptible to diabetes at BMI levels considered healthy for European-descent populations (Hsu et al., 2015). This “metabolically obese normal-weight” phenotype occurs due to a tendency to accumulate visceral fat without a corresponding increase in overall body mass (Ding et al., 2016). Relying on universal BMI thresholds masks the actual metabolic danger for Asian Americans, highlighting an urgent need for demographic-specific risk evaluations.

Beyond demographics, diabetes rates across the United States exhibit clear geographic clustering, indicating that macro-level environments independently impact metabolic health (Dugani et al., 2022). The “obesogenic environment” framework suggests that community behavioral norms, limited preventive care access, and socioeconomic hardships elevate a population's overall risk (Iguacel et al., 2025). Despite this, many epidemiological studies rely on single-level regressions, blurring compositional (individual) and contextual (state-level) effects. Multilevel modeling is essential to properly separate these variances and explore cross-level interactions, such as evaluating whether a high-risk state environment amplifies an individual's physical vulnerabilities (Diez-Roux, 2000).

Furthermore, analyzing cross-sectional data requires careful handling of differential mortality, or survival bias. Severe behaviors like heavy alcohol use and chronic smoking sometimes show paradoxical, inverse relationships with age-related diseases like diabetes (Flood et al., 2025). Because heavy smokers or binge drinkers frequently die prematurely, those surviving into older adulthood constitute a highly selected, metabolically resilient subgroup (Campagna et al., 2019). Failing to account for this age-related survivor effect risks the incorrect conclusion that damaging habits provide biological protection.

Addressing these empirical gaps, this study leverages nationally representative data from 406,782 adults via the 2024 Behavioral Risk Factor Surveillance System (BRFSS) (Rolle-Lake and Robbins, 2025). Applying a robust multilevel logistic regression approach grounded in the Socio-Ecological Model of health, this research pursues four objectives: (1) to partition diabetes variance to weigh state-level contextual impacts versus individual risk factors; (2) to clarify racial and ethnic disparities, emphasizing hidden risk profiles of Asian Americans; (3) to examine cross-level interactions between state-level obesity and individual adiposity; and (4) to use age-stratified analyses to isolate survival bias effects concerning smoking and binge drinking.

## 2 Methods

### 2.1 Study design and population

This cross-sectional study utilized secondary data from the 2024 BRFSS (Nelson et al., 2001). The initial starting sample included 457,670 respondents. After excluding respondents younger than 18 years, individuals with missing data for diabetes status, and women reporting only gestational diabetes, the final analytical sample comprised 406,782 adults nested within 53 states and United States territories.

### 2.2 Measures

The dependent variable was self-reported physician-diagnosed diabetes, operationalized as a binary outcome (yes/no). Specifically, respondents reporting “no, pre-diabetes or borderline diabetes” were coded as 0 (non-diabetic), while those reporting “yes, but only during pregnancy” (gestational diabetes) were excluded from the analysis to prevent confounding. Level 1 (individual-level) predictors included age, sex, race/ethnicity (categorized as Non-Hispanic White [reference group], Non-Hispanic Black, Hispanic, Asian, and Other), education, income, obesity status, current smoking status, physical inactivity, binge drinking, self-rated poor general health, and clinical diagnoses of depression, arthritis, and heart disease. To isolate purely within-state associations, continuous individual-level variables were group-mean centered. Level 2 (state-level) predictors were derived directly by aggregating the 2024 BRFSS individual data into state-specific proportions for obesity, smoking, poverty, and the urbanicity proportion. All Level 2 variables were grand-mean centered to reduce multi-collinearity and facilitate interpretation of the intercept.

### 2.3 Statistical analysis

Given the hierarchical data structure (with individuals nested within states), multilevel logistic regression models were fitted using Generalized Linear Mixed Models estimated via Laplace approximation (Breslow and Clayton, 1993). Four sequential models were constructed. First, a null (intercept-only) model was used to estimate the baseline between-state variance, from which the Intraclass Correlation Coefficient (ICC) was calculated on the latent variable scale as:

$$\mathrm{ICC}=\frac{\tau_{00}}{\tau_{00}+\frac{\pi^{2}}{3}}$$

Second, a Model 1 examined individual-level predictors. Third, a fully adjusted multilevel model (Model 2) incorporated state-level predictors. Fourth, a cross-level interaction model (Model 3) tested whether state-level obesity prevalence moderated the effect of individual obesity on diabetes risk. As noted, missing observations were handled strictly through complete case analysis, ensuring that all models were fitted using identical, fully completed records to maintain structural consistency across model transitions. Analyses were performed in R using the glmer function within the lme4 package, utilizing the bobyqa optimizer to achieve model convergence.

### 2.4 Survival bias testing

To investigate potential survival bias, which may erroneously manifest as spurious and biologically implausible “protective” effects for high-risk behaviors such as current smoking and binge drinking, an age-stratified analysis was conducted. Multilevel logistic regression models were fitted independently for each of three distinct age strata: young adults (18–34 years), middle-aged adults (35–54 years), and older adults (≥55 years). This stratification enabled the evaluation of the longitudinal trajectory of Odds Ratios (ORs) across the lifespan within a cross-sectional framework. The threshold for statistical significance in all tests was set at α=0.05.

### 2.5 Ethical compliance

The BRFSS is a publicly available, de-identified dataset administered by the Centers for Disease Control and Prevention. Therefore, this study was exempt from institutional review board approval and the requirement for informed consent.

## 3 Results

### 3.1 Sample characteristics and individual-level predictors

Of the 406,782 adults in the analytical sample, 59,070 (crude prevalence: 14.5%) reported physician-diagnosed diabetes. Compared with unaffected individuals, respondents with diabetes were significantly older (mean age: 63.9 versus 53.4 years), predominantly male (51.7% versus 49.1%), had lower socioeconomic status, and exhibited markedly higher rates of obesity (52.1% versus 30.4%), physical inactivity (37.2% versus 20.1%), poor self-rated health (40.3% versus 15.9%), clinical depression (25.5% versus 20.6%), arthritis (54.0% versus 31.7%), and heart disease (22.3% versus 7.5%) (all *p* < 0.01). Conversely, binge drinking was paradoxically less common among individuals with diabetes (6.1% versus 14.1%, *P* < 0.01). Racial/ethnic distributions also differed significantly between groups (*P* < 0.01) (Table 1).

**Table 1:** Baseline demographic and clinical characteristics stratified by diabetes status among United States adults (*N* = 406,782), behavioral risk factor surveillance system, 2024.

Characteristic		No diabetes (*n* = 347,712)	Diabetes (*n* = 59,070)	*p*-value
Age, years, mean (SD)		53.4 (17.3)	63.9 (11.2)	<0.01
Male sex, n (%)		170,746 (49.1)	30,539 (51.7)	<0.01
Race/ethnicity, n (%)				<0.01
	White	261,172 (75.1)	42,353 (71.7)	
	Black	24,340 (7.0)	6439 (10.9)	
	Hispanic	35,119 (10.1)	5612 (9.5)	
	Asian	9736 (2.8)	1240 (2.1)	
	Other	17,386 (5.0)	3426 (5.8)	
Education score, mean (SD) †		0.03 (0.98)	−0.17 (1.01)	<0.01
Income score, mean (SD) ‡		0.07 (2.13)	−0.41 (2.32)	<0.01
Obese (BMI ≥ 30 kg/m^2^), n (%)		105,704 (30.4)	30,770 (52.1)	<0.01
Current smoker, n (%)		37,553 (10.8)	6675 (11.3)	<0.01
No physical activity, n (%)		69,890 (20.1)	21,974 (37.2)	<0.01
Binge drinker, n (%) §		49,027 (14.1)	3603 (6.1)	<0.01
Poor general health, n (%)		55,286 (15.9)	23,805 (40.3)	<0.01
Depression, n (%)		71,629 (20.6)	15,063 (25.5)	<0.01
Arthritis, n (%)		110,225 (31.7)	31,898 (54.0)	<0.01
Heart disease, n (%) ‖		26,078 (7.5)	13,173 (22.3)	<0.01

Note: SD, standard deviation. *P*-values were derived from independent *t*-tests for continuous variables and chi-square tests for categorical variables.

† Education score: standardized variable derived from 4-level education categories (1 = less than high school, 4 = college graduate), centered at the sample mean.

‡ Income score: standardized variable derived from 7-level household income categories (1 ≤$10,000, 7 = ≥$200,000), centered at the sample mean.

§ Binge drinking: ≥5 alcoholic drinks on one occasion for men, ≥4 for women during the past 30 days.

¶ Poor general health: self-rated health status as “fair” or “poor” (reference: excellent, very good, or good).

‖ Heart disease: self-reported history of myocardial infarction or coronary heart disease.

Table 2 presents the results of the multilevel logistic regression model adjusting for individual-level factors (Model 1). Obesity emerged as the strongest modifiable risk factor for diabetes (OR=2.33; 95%CI: 2.28,2.38), followed closely by poor general health (OR=2.18; 95%CI: 2.13,2.23). Marked racial and ethnic disparities emerged: Asian adults manifested the highest diabetes risk (OR=2.05; 95%CI: 1.91,2.20), followed by Black (OR=1.76; 95%CI: 1.70,1.82) and Hispanic adults (OR=1.54; 95%CI: 1.48,1.60), relative to their Non-Hispanic White counterparts. Incremental shifts in higher education (OR=0.93 per level; 95%CI: 0.92,0.94) and household income (OR=0.97 per level; 95%CI: 0.96,0.97) exhibited independent protective properties. Unexpectedly, current smoking (OR=0.89; 95%CI: 0.86,0.92) and binge drinking (OR=0.62; 95%CI: 0.60,0.65) exhibited inverse associations with diabetes odds—findings that hypothesized a severe structural confounding effect, which we subsequently evaluated using age-stratified analyses to isolate potential survival bias (Table 2).

**Table 2:** Multilevel logistic regression results for individual-level predictors of diabetes among United States adults (*N* = 406,782), behavioral risk factor surveillance system, 2024.

Predictor		OR (95% CI)
Age (per year)		1.05 (1.04, 1.05)
Male (vs female)		1.36 (1.34, 1.39)
Race/ethnicity (ref: White)		
	Black	1.76 (1.70, 1.82)
	Hispanic	1.54 (1.48, 1.60)
	Asian	2.05 (1.91, 2.20)
	Other	1.49 (1.42, 1.55)
Education (per level)		0.93 (0.92, 0.94)
Income (per level)		0.97 (0.96, 0.97)
Obesity		2.33 (2.28, 2.38)
Current smoking		0.89 (0.86, 0.92)
Physical inactivity		1.36 (1.33, 1.39)
Binge drinking		0.62 (0.60, 0.65)
Poor general health		2.18 (2.13, 2.23)
Depression		1.24 (1.21, 1.27)
Arthritis		1.20 (1.18, 1.23)
Heart disease		1.70 (1.66, 1.74)

CI, confidence interval; OR, odds ratio. Models adjusted for all predictors simultaneously with random intercepts for states (*n* = 53). All continuous predictors were group-mean centered.

### 3.2 State-level contextual effects and survival bias analysis

The baseline intercept-only multilevel model revealed significant between-state variance in diabetes risk ($\tau_{00}=0.054$, p<0.01). The calculated latent variable ICC was 0.014, indicating that 1.4% of the total variance in diabetes diagnosis was directly attributable to contextual differences between states.

Incorporating macro-level contextual factors into the model (Model 2) reduced the residual between-state variance from $\tau_{00}=0.054$ (null model) to $\tau_{00}=0.023$ (full model), representing a 57.4% proportional variance reduction. This indicates that macro-level environmental characteristics explain more than half of the geographic variation in diabetes risk. After adjusting for individual-level factors, state-level obesity prevalence remained a significant independent predictor of diabetes: a 10-percentage-point increase in macro-level obesity rate was associated with a 38% increase in individual diabetes odds (OR=1.38; 95%CI: 1.17,1.63). In contrast, state-level smoking rates (OR=5.63; 95%CI: 0.14,22.34), state poverty rates (OR=5.36; 95%CI: 0.48,59.8), and the proportion of residents living in metropolitan counties (OR=1.07; 95%CI: 0.82,1.41) did not display statistically significant independent associations with the outcome.

In Model3, the interaction term between individual-level obesity and state-level obesity prevalence was statistically significant on the log-odds scale (β=−0.754, 95%CI: −1.287,−0.221). This negative interaction parameter indicates that the individual-level effect of obesity on diabetes risk is partially attenuated in states with higher overall obesity prevalence. Specifically, the odds ratio for individual obesity was 2.34 (95%CI, 2.29,2.39) in states exhibiting mean obesity prevalence, but experienced a significant slope dampening when evaluated within contexts featuring higher aggregated macro-level adiposity. (Table 3)To test the empirical validity of the unexpected inverse associations observed between baseline behavioral habits and diabetes risk in our fully adjusted models, we executed an independent age-stratified analytical protocol. For current smoking, the observed association was entirely null among young adults aged 18–34 years (OR=1.00; 95%CI: 0.83,1.19). However, tracking this point estimate across progressively older age cohorts revealed a systematic, downward shift in the odds ratios. In middle-aged adults aged 35–54 years, current smoking manifested a significant inverse relationship (OR=0.91; 95%CI: 0.85,0.97), which culminated in the maximum negative deviation observed among older adults aged ≥55 years(OR=0.80; 95%CI: 0.77,0.83). A nearly identical, age-dependent trajectory emerged for binge drinking: the inverse association was weakest and closest to the null in the youngest cohort (OR=0.87; 95%CI: 0.75,1.00), moderately strengthened in middle-aged adults (OR=0.65; 95%CI: 0.61,0.69), and became highly pronounced among older adults (OR=0.57; 95%CI: 0.54,0.60). (Table 4).

**Table 3:** Multilevel logistic regression results for state-level predictors and cross-level interaction for diabetes risk among United States adults (*N* = 406,782), behavioral risk factor surveillance system, 2024.

Predictor		Odds ratio/coefficient	95% CI
Model 2: Main State-Level Effects			
	State Obesity Prevalence (per 10% increase)	1.38	1.17, 1.63
	State Smoking Rate	5.63	0.14, 22.34
	State Poverty Rate	5.36	0.48, 59.80
	State Urbanicity Proportion	1.07	0.82, 1.41
Model 3: Cross-Level Interaction			
	Individual Obesity × State Obesity (β)	−0.754	−1.287, −0.221

Note: All state-level predictors are grand-mean centered. The individual obesity variable was group-mean centered. Confidence intervals (CI) are derived from the fully adjusted multilevel logistic regression models.

**Table 4:** Age-stratified multilevel logistic regression results for behavioral risk factors and diabetes risk among United States adults (*N* = 406,782), behavioral risk factor surveillance system, 2024.

Behavioral predictor	Young adults (18–34 years), OR (95% CI)	Middle-aged adults (35–54 years), OR (95% CI)	Older adults (≥55 years), OR (95% CI)
Current smoking	1.00 (0.83, 1.19)	0.91 (0.85, 0.97)	0.80 (0.77, 0.83)
Binge drinking	0.87 (0.75, 1.00)	0.65 (0.61, 0.69)	0.57 (0.54, 0.60)

Note: CI, confidence interval; OR, odds ratio. Models were fitted independently within each age stratum and adjusted for sex, race/ethnicity, education, income, obesity, physical inactivity, poor general health, depression, arthritis, heart disease, and state-level random intercepts.

## 4 Discussion

In this nationally representative multilevel analysis, we demonstrated that state-level obesogenic environments independently amplify diabetes risk, while also unmasking survival bias in behavioral risk factors and highlighting the distinct metabolic vulnerability of Asian Americans.

Geographic heterogeneity in diabetes risk persisted after adjusting for individual-level covariates. Southeastern states (e.g., Mississippi, Louisiana, Alabama, West Virginia) exhibited higher-than-predicted diabetes risk, indicating that residents face elevated odds beyond their individual profiles. Conversely, Northeastern and Western states demonstrated lower-than-predicted risk. This patterning implies that unmeasured macro-level factors, such as healthcare quality, food environments, and public health policies, meaningfully influence diabetes risk independently of individual composition. This variation underscores the critical need for place-based, contextually targeted interventions (Hill et al., 2013; Siegel and Albright, 2021). Consequently, “positive deviant” states outperforming epidemiological expectations could serve as blueprints for scalable prevention strategies (Fig. 1).

**Fig. 1:**
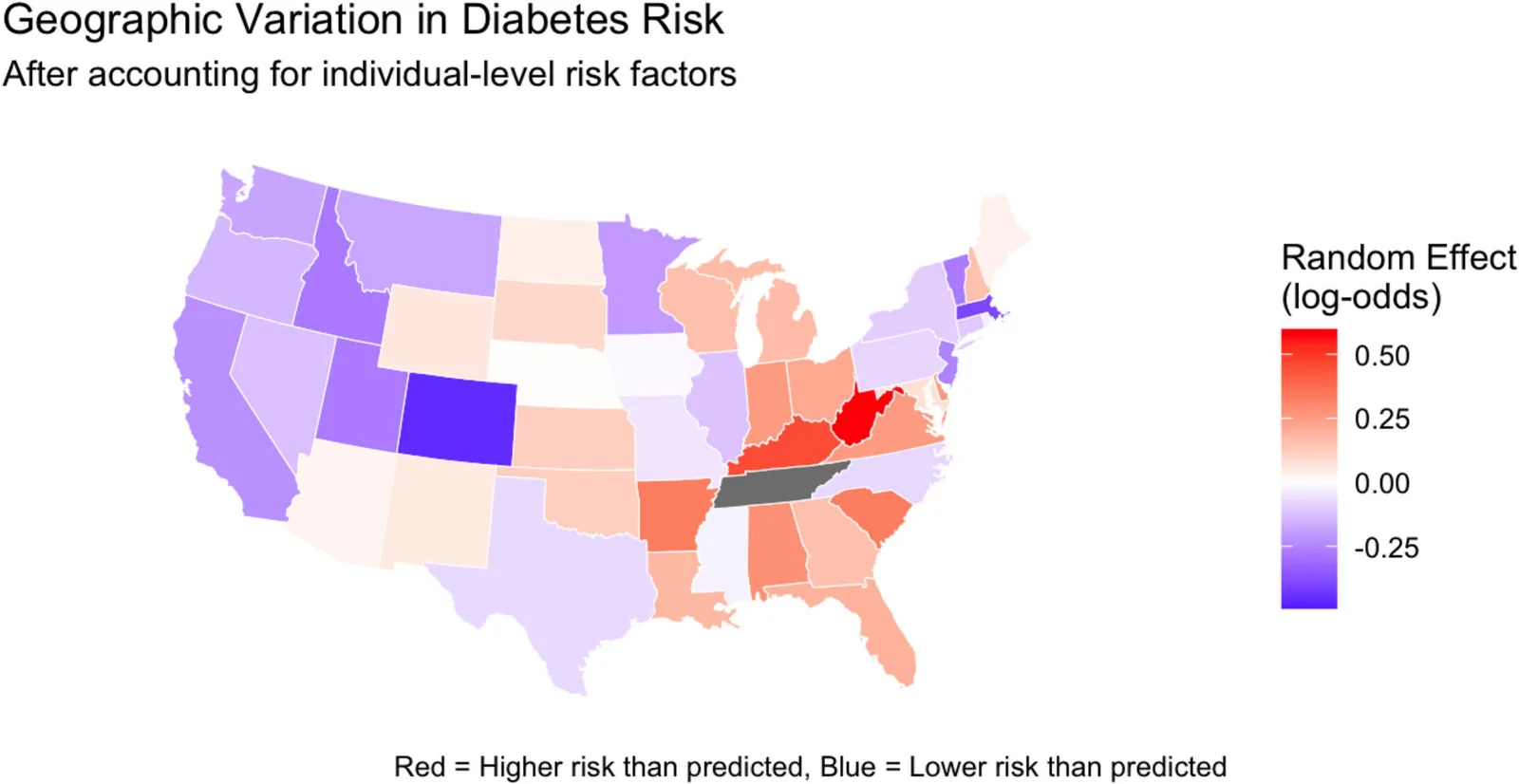
Geographic variation in state-level residual diabetes risk after adjustment for individual-level factors among United States adults (*N* = 406,782), behavioral risk factor surveillance system, 2024.

Age-stratified analyses revealed that the unexpected inverse associations of smoking and binge drinking with diabetes strengthened with advancing age— a classic signature of survival bias (Hernán et al., 2004). The lack of protective effects in younger adults strongly refutes any underlying biological mechanism. Instead, this trend captures differential mortality. Individuals with heavy smoking or drinking habits experience elevated premature death rates from cardiovascular, respiratory, and hepatic diseases, alongside various cancers (Committee, 2024). Because these conditions often cause mortality before common diabetes onset, surviving older smokers and heavy drinkers represent a highly selected population whose risk profile does not reflect the originally exposed cohort (Doll et al., 2004).

This phenomenon aligns with prior epidemiologic literature demonstrating that apparent protective associations attenuate after accounting for selective survival. Similar patterns appear in studies of smoking and Alzheimer's disease (Hernán et al., 2008). and parallel the “obesity paradox” in cardiovascular disease, where selective survival, reverse causation, and frailty are major explanatory factors (Banack and Kaufman, 2014). Beyond survival bias, reverse causality likely contributes to this finding. Because our reference group combined “former” and “never” smokers, individuals quitting smoking post-diagnosis inflated diabetes prevalence among non-smokers, spuriously making current smoking appear protective. Consequently, our findings carry important clinical implications: smoking and heavy alcohol use must not be interpreted as protective against metabolic disorders (Willi et al., 2007). These observed inverse associations in cross-sectional data reflect survivor selection and methodological biases rather than causal biological benefits. Clinicians should continue counseling patients on the well-established harms of smoking and excessive alcohol use, including increased all-cause mortality and chronic disease risks (Bryazka et al., 2022).

A second major insight is the elevated diabetes risk among Asian American adults. After adjusting for covariates, Asian adults demonstrated more than double the odds of diabetes compared with White adults (OR = 2.05). This disparity exceeded the risk among Black and Hispanic adults, highlighting an often underrecognized pattern in the United States metabolic health disparities. Prior studies explain this through a metabolically high-risk phenotype: compared with populations of European ancestry, individuals of Asian descent frequently exhibit greater visceral adiposity and earlier metabolic dysfunction at comparable body weights, thereby developing diabetes at substantially lower BMI thresholds (J. C. Chan et al., 2009; Lee et al., 2011; Narayan and Kanaya, 2020).

However, ethnicity-specific BMI threshold use remains inconsistent in routine United States clinical practice, potentially contributing to the underrecognition of diabetes risk among Asian American populations (Diabetes⁎, 2025b).

Currently, the American Diabetes Association recommends baseline screening for all adults aged 35 years and older, yet these guidelines do not uniformly mandate ethnicity-specific BMI thresholds (Diabetes⁎, 2025a). Our findings strongly suggest that initiating screening for Asian Americans at lower BMI cutoffs, or potentially at younger ages, could drastically improve early detection and prevention (Hsu et al., 2015). Incorporating state-level predictors (Model 2) reduced between-state variance by 57%, suggesting contextual factors explain substantial geographic variation in diabetes risk. State-level obesity prevalence independently predicted diabetes risk after adjusting for individual BMI (OR = 1.38; 95% CI: 1.17, 1.63 per 10-percentage-point increase), indicating higher-obesity environments contribute to risk beyond individual adiposity. Because state-level obesity likely captures broader environmental and structural influences, this association should not be interpreted as a direct contextual effect of obesity itself (Diez Roux, 2001).

Notably, a significant cross-level interaction between individual and state-level obesity indicated that the individual obesity-diabetes association attenuated in states with higher background obesity prevalence. Several mechanisms may explain this moderation. First, in environments with widespread obesity, this moderation may stem from broader contextual determinants (e.g., dietary environments, physical activity infrastructure, healthcare access) may overwhelm individual risk and contribute relatively more to diabetes risk than individual adiposity. Second, overwhelming individual risk, differential screening practices could increase case detection among lower-risk individuals, reducing apparent between-group differences. Finally, this attenuation may reflect groups, or statistical compression from resulting from reduced exposure contrast (range restriction), wherein highly prevalent exposures exhibit smaller observable effect estimates.

Our individual-level findings align with established epidemiological evidence identifying excess adiposity as the dominant modifiable risk factor for type 2 diabetes (Hu et al., 2001). Similarly, protective associations for higher income and educational attainment align with the social determinants of health framework, recognizing socioeconomic conditions as fundamental drivers of cardiometabolic outcomes (Marmot, 2020).

Although elevated metabolic risk among Asian Americans is gaining recognition, it remains underrepresented in the United States public health discourse compared with disparities traditionally emphasized among Black and Hispanic populations (Cheng et al., 2019). Our findings reiterate that relying on conventional BMI thresholds underestimates diabetes susceptibility among Asian adults and obscures clinically meaningful risk among individuals with normal-range body weight (J. C. N. Chan et al., 2009).

Furthermore, the significant cross-level interaction between individual and state obesity is a novel finding warranting independent replication. If confirmed in future longitudinal cohorts, these results suggest the metabolic consequences of excess adiposity are context-dependent. This implies that environmental and population-level interventions must complement individualized weight-management approaches to effectively reduce the diabetes burden (Rubino et al., 2025; Swinburn et al., 2019). Strengths include a nationally representative dataset ensuring robust statistical power and generalizability across the United States. Advanced multilevel modeling rigorously disentangles individual-level attributes from macro-environmental effects, avoiding common ecological fallacies. Furthermore, our age-stratified approach unmasks survival bias, providing a crucial methodological corrective for behavioral data regarding smoking and alcohol consumption associations. Lastly, highlighting the Asian American metabolic vulnerability challenges reliance on universal BMI thresholds, advocating for equitable, ethnicity-specific screening guidelines.

Several limitations warrant consideration. The cross-sectional design inherently precludes causal inference, and unmeasured confounding or self-report misclassification bias remains. As revealed, survival bias likely explains the inverse associations with smoking and binge drinking, highlighting the need to interpret cross-sectional behavioral data cautiously. Additionally, missing data excluded incomplete records in the complete case analysis, potentially affecting generalizability. Finally, the age variable relies on imputed category midpoints rather than exact continuous years, though this remains standard practice in large surveillance platforms.

## 5 Conclusions

In this multilevel analysis of United States adults, individual obesity remains the most critical modifiable risk factor for diabetes. However, substantial geographic variation exists, and state-level obesogenic environments independently amplify this risk. Demographically, Asian Americans face the highest relative odds of diabetes, emphasizing an urgent need for targeted screening using ethnicity-specific BMI screening thresholds. Methodologically, our analyses definitively show that apparent protective effects of smoking and heavy drinking are artifacts of survival bias, not true biological phenomena. Moving forward, clinical practices must adjust risk profiling for vulnerable populations, while public health strategies must target both individual behaviors and the macro-environments sustaining them.

## CRediT authorship contribution statement

**Masoud Imani:** Writing – review & editing, Writing – original draft, Visualization, Validation, Formal analysis, Data curation. **Seyedshayan Shojaei:** Writing – review & editing, Resources. **Kimia Heidari:** Writing – review & editing, Supervision. **Mohammad Javad Shabani:** Writing – review & editing, Writing – original draft, Methodology, Data curation. **Hamid Mohsenabadi:** Writing – review & editing, Supervision, Investigation. **Bagher Larijani:** Writing – review & editing, Software, Resources. **Mohammad Reza Mohajeri-Tehrani:** Writing – review & editing, Resources. **Amir Kasaeian:** Writing – review & editing, Writing – original draft, Supervision, Project administration, Formal analysis.

## Ethics approval

The data are publicly available and the secondary analyses were conducted on anonymous data.

## Ethics declaration

### Informed consent and patient details

Written informed consent to take part in the study and to publish the article has been obtained from all participants or their legal representatives. The privacy rights of participants have been observed.

### Studies in human

This study was performed in compliance with relevant laws, regulatory frameworks and guidelines where the research took place.

Ethics committee approval was not required under relevant laws and institutional guidelines. Data were gathered by CDC. So this study is secondary analysis and the manuscript do not need new any committee approval.

## Funding

This research did not receive any specific grant from funding agencies in the public, commercial, or not-for-profit sectors.

## Declaration of competing interest

The authors declare that they have no known competing financial interests or personal relationships that could have appeared to influence the work reported in this paper.

## Data Availability

Data will be made available on request.
